# The association of perceived stress on disordered eating among female college students: the mediating role of negative emotions and the moderating role of physical exercise

**DOI:** 10.3389/fnut.2026.1935263

**Published:** 2026-09-14

**Authors:** Xiangli Ni, Xiaojuan Zhu

**Affiliations:** Physical Education College, Jiangxi Normal University, Nanchang, China

**Keywords:** emotional eating, negative emotions, perceived stress, physical exercise, restrained eating

## Abstract

**Background:**

Disordered eating is prevalent among female college students and is associated with adverse physical and mental health outcomes. This study examined the mediating role of negative emotions in the relationship between perceived stress and three types of disordered eating (emotional eating, restrained eating, and external eating), as well as the moderating role of physical exercise among female college students.

**Methods:**

A cross-sectional survey was conducted among 604 female college students from four universities in China. Participants completed validated measures assessing perceived stress, negative emotions, physical exercise, and disordered eating. Mediation and moderation analyses were performed using the PROCESS macro (Model 4 and Model 59) with 5,000 bootstrap resamples.

**Results:**

Perceived stress was positively associated with all three types of disordered eating. Negative emotions partially mediated the relationship between perceived stress and emotional eating, and fully mediated the relationship with restrained eating, but did not mediate the relationship with external eating. Physical exercise negatively moderated the associations between negative emotions and both emotional eating and restrained eating. However, physical exercise positively moderated the relationship between perceived stress and negative emotions, though with very small effect sizes.

**Conclusion:**

Negative emotions serve as a key mechanism linking perceived stress to emotionally driven eating patterns, with differential mediating roles across eating behavior types. Physical exercise may serve as a downstream buffer that disrupts the translation of negative affect into maladaptive eating behaviors. These findings highlight the need for targeted interventions that address distinct pathways for different types of disordered eating among female college students.

## 1 Introduction

Disordered eating refers to a subclinical symptom of eating disorders triggered by weight control efforts, negative emotional responses, or external stimuli (1). Disordered eating is classified as restrictive eating, emotional eating, and external eating (1). Although this condition does not fully meet the clinical diagnostic criteria for eating disorders, such maladaptive eating behaviors are adversely associated with individuals’ physical and mental health (1), with female college students being a high-risk group for these behaviors (2). Research has found that the detection rate of disordered eating among female college students is 5.11% (2). Particularly under the influence of internet media promoting the “thinness equals beauty” ideal for women, female college students not only pay greater attention to their body shape and restrict their diet but also experience appearance anxiety (3). These behaviors and anxieties may be potentially related to disordered eating. Therefore, investigating the correlates of disordered eating among female college students is of particular importance.

### 1.1 The association between perceived stress and disordered eating

Perceived stress refers to an individual’s subjective perception of internal and external stressful events, which may relate to their cognitive, emotional, and physiological states (4). Some scholars suggest that excessive and prolonged stress may be accompanied by physiological dysregulation and an “allostatic” psychological state (5), such as endocrine disorders (5), emotional exhaustion (6), or behavioral abnormalities (7). Empirical studies have demonstrated that under acute stress, individuals increase their intake of sweet foods (8). Research has found that college students experiencing excessive stress are more likely to report emotional eating (9). Other studies have indicated that under excessive stress, both snack and meal consumption decrease significantly (10). Collectively, these findings suggest that excessive stress may be associated with individuals’ disordered eating. Based on the above evidence, we propose the following hypothesis: H1: Perceived stress is associated with disordered eating among female college students.

### 1.2 The mediating role of negative emotions

Currently, research on the psychological mechanisms through which perceived stress is related to disordered eating among female college students remains relatively scarce, warranting further in-depth investigation. Negative emotions may play a mediating role between the two variables. On the one hand, perceived stress is often accompanied by negative emotions. According to Arnold’s “appraisal--excitation” theory, individuals engage in perceptual analysis of stressful events in the cerebral cortex, form a negative evaluation (e.g., perceiving the event as frightening), which subsequently arouses negative emotions (11). Research has also indicated that stress is a significant correlate of negative emotions among female college students (12). On the other hand, negative emotions may be related to disordered eating. In daily life, being in a negative emotional state tends to undermine one’s cognitive capacity for regulating eating and impairs sound food choices. Yet, the actual responses vary greatly across individuals: while some exhibit overeating behaviors (13), others react by refusing to eat or significantly reducing their food intake (14). Although external eating is primarily characterized by heightened sensitivity to external food cues, negative emotions may increase individuals’ susceptibility to external food stimuli by altering attentional biases and impulse control (15). Relevant studies have also shown that negative emotions are positively correlated with disordered eating among adolescents (16). Furthermore, research has found that perceived stress, negative emotions, and disordered eating are closely interrelated (17). Based on previous theoretical and empirical foundations, this study proposes the research hypothesis H2: Negative emotions mediate the relationship between perceived stress and disordered eating among female college students.

### 1.3 The moderating role of physical exercise

Furthermore, physical exercise may play a moderating role in the relationship between perceived stress and disordered eating. Physical exercise refers to purposeful, planned fitness and recreational physical activities conducted during individuals’ leisure time, primarily encompassing three elements: intensity, duration, and frequency (18). As a positive self-regulatory strategy for physical and mental health, physical exercise has a certain alleviative association with individuals’ perceived stress (19). According to the “stress-buffering model” proposed by Cohen (20), when individuals potentially perceive stressful events as stress, physical exercise can suppress the adverse psychological and physiological reactions that are related to perceived stress through physiological arousal (21). Therefore, physical exercise plays the following moderating roles in the mediation model of this study: First, physical exercise may moderate the relationship between perceived stress and disordered eating. Based on the stress-buffering model, the physiological disturbances (e.g., elevated endocrine levels) and disordered behaviors (e.g., emotional eating) associated with perceived stress may be mitigated through physical exercise (21), thereby attenuating the stress-related disordered eating. Although direct research on the moderating effect of physical exercise in the “stress-eating” relationship remains insufficient, existing evidence has indicated that regular physical activity can buffer the negative impact of daily stress on individuals’ dietary preferences (22). Second, physical exercise may moderate the relationship between perceived stress and negative emotions. Relevant studies have suggested that physical exercise is associated with improvements in endocrine disorders and negative psychological states, thereby being linked to reduced adverse emotions (e.g., depression and anxiety) that are related to stress (23). Third, physical exercise may moderate the relationship between negative emotions and disordered eating. As a “byproduct” of perceived stress, negative emotions are often accompanied by enhanced physiological arousal and depletion of self-regulatory resources, thereby being associated with an increased risk of disordered eating (21). Physical exercise may be associated with reduced disordered eating that is related to negative emotions by enhancing individuals’ bodily awareness and sense of self-control (24). Previous research has indicated that physical exercise is associated with reduced anxiety levels among college students (19), thereby being associated with improved eating-related psychological distress (25), suggesting that physical exercise may serve as an important protective factor against negative emotions and their associated disordered eating. Based on the above theoretical and empirical foundations, this study proposes Hypothesis H3: Physical exercise moderates the relationships between perceived stress and negative emotions, between perceived stress and disordered eating, and between negative emotions and disordered eating.

## 2 Participants and methods

A convenience sampling method was employed to recruit female college students from 14 classes across Grades 1 to 3 at four target universities in China. It should be noted that the 14 classes were not evenly distributed across the four universities (i.e., the class counts were 4, 4, 3, and 3, respectively), as the distribution was determined by each institution’s academic scheduling and the availability of eligible classes. The recruited classes covered all three academic years. To align with the study’s focus on female college students, only classes composed exclusively of female students (i.e., single-sex classes) were selected for recruitment. This single-sex class structure is a common administrative arrangement in certain Chinese universities, which facilitated the recruitment of a homogeneous female sample without the need for within-class gender-based exclusion. A total of 700 students from these classes voluntarily participated in the anonymous survey. Criteria for invalid responses included failure to answer all questions and patterned responding, such as identical scores across all items or regular response sequences (e.g., 1, 2, 3, 4, 5, 1, 2, 3, 4, 5, 1, 2, 2, 3, 4, 5). After excluding invalid questionnaires, 604 valid responses were retained from the initial 700, yielding an effective response rate of 86.29%. Participants ranged in age from 17 to 23 years. The demographic characteristics of the participants are presented in Table 1. The study was approved by the Ethics Committee of Physical Education College, Jiangxi Normal University (Approval No. [IRB-JXNU-PEC-2025371]). Written informed consent was obtained from all participants prior to their involvement. All procedures were conducted in accordance with the Declaration of Helsinki.

**Table 1:** Demographic characteristics.

Variable	Category	*N*	Percentage (%)	Cumulative percentage (%)
Residence	Urban	357	59.1	59.1
	Rural	247	40.9	100
Grade	Freshmen	228	37.7	37.7
	Sophomore	183	30.3	68
	Junior	193	32.0	100
Major	Sciences and Engineering	247	40.9	40.9
	Humanities	113	18.7	59.6
	Social Sciences	160	26.5	86.1
	Arts and Physical Education	84	13.9	100

### 2.1 Measures

#### 2.1.1 Perceived stress scale

Perceived Stress Scale (PSS), originally developed by Cohen et al. (26) and revised by Yang and Huang (27), was employed in this study. The revised PSS has been widely applied among Chinese adult populations and has demonstrated satisfactory reliability and validity (27). Therefore, this study adopted the revised PSS to assess the level of perceived stress among female college students in specific contexts. The scale consists of 10 items, rated on a 5-point Likert scale ranging from 1 (strongly disagree) to 5 (strongly agree). The total score was calculated by summing all items, with higher average scores indicating greater levels of perceived stress. Confirmatory factor analysis (CFA) yielded acceptable fit indices for the perceived stress scale in this study: CFI = 0.980, TLI = 0.955, RMSEA = 0.075, SRMR = 0.061. The Cronbach’s alpha coefficient was 0.753.

#### 2.1.2 Negative emotions scale

Negative Emotions Scale was originally compiled by Watson et al. (28) and subsequently revised by Huang et al. (29). The revised scale has demonstrated good reliability and cultural applicability in Chinese sample studies (29). The scale comprises 10 items, rated on a 5-point Likert scale ranging from 1 (strongly disagree) to 5 (strongly agree). The total score was calculated by summing all items, with higher average scores indicating higher levels of negative emotions. Confirmatory factor analysis (CFA) yielded acceptable fit indices for negative emotions scale in this study: CFI = 0.975, TLI = 0.954, RMSEA = 0.075, SRMR = 0.034. The Cronbach’s alpha coefficient was 0.901.

#### 2.1.3 Physical exercise rating scale

Physical Exercise Rating Scale, revised by Liang et al. (30), was used to assess physical exercise levels. This scale evaluates physical exercise from three dimensions: exercise intensity, frequency, and duration. Each dimension is scored on a 1–5-point scale, and the total physical exercise score was calculated using the formula: Physical exercise volume = exercise intensity × (exercise duration – 1) × exercise frequency. The criteria for classifying physical exercise volume were as follows: scores ≤ 19 indicated low volume, scores between 20 and 42 indicated moderate volume, and scores ≥ 43 indicated high volume. This scale has demonstrated good reliability among college student populations (2).

#### 2.1.4 Disordered eating scale

Disordered Eating Scale, developed by Qin et al. (31), was adopted in this study. This scale has demonstrated satisfactory reliability and cultural applicability in Chinese sample studies (31). The scale comprises three subscales: emotional eating (5 items), restrained eating (5 items), and external eating (5 items). All items are rated on a 5-point Likert scale ranging from 1 (strongly disagree) to 5 (strongly agree). Confirmatory factor analysis (CFA) yielded acceptable fit indices for disordered eating scale in this study: CFI = 0.943, TLI = 0.931, RMSEA = 0.073, SRMR = 0.059. The Cronbach’s alpha coefficients for the three subscales were 0.927, 0.845, and 0.744, respectively.

### 2.2 Data analysis

Data analyses were conducted using SPSS 26.0. First, reliability testing and confirmatory factor analysis (CFA; performed using Mplus 8.3) were conducted to assess the properties of all scales. Given that all data were collected through self-report questionnaires, Harman’s single-factor test was conducted to examine potential common method bias; the unrotated principal component factor analysis revealed that eight factors had initial eigenvalues greater than 1, and the first factor accounted for 23.755% of the total variance (< 40% threshold), indicating that common method bias was not a serious concern. Next, descriptive statistics and Pearson correlation analysis were performed. Finally, the PROCESS macro (Version 3.5) developed by Hayes was employed. Model 4 was used to test the mediating effect of negative emotions, with perceived stress as the independent variable and the three disordered eating subtypes (emotional eating, restrained eating, and external eating) as separate dependent variables. Model 59 was then utilized to examine the moderated mediating effects. All analyses employed the bootstrap method with 5,000 resamples to estimate 95% confidence intervals.

## 3 Results

### 3.1 Descriptive statistics and correlation analysis

As shown in Table 2, perceived stress was significantly positively correlated with all three dimensions of disordered eating. Negative emotions were significantly positively correlated with perceived stress and all three dimensions of disordered eating. Physical exercise was significantly negatively correlated with perceived stress and negative emotions. Additionally, physical exercise was significantly negatively correlated with emotional eating but significantly positively correlated with restrained eating and external eating. Demographic variables showed significant correlations with various study variables and were therefore included as control variables in subsequent regression models for further testing. Additionally, the absolute values of skewness and kurtosis for all variables were below 2 and 7, respectively, indicating that the data approximately followed a normal distribution and were appropriate for subsequent analyses.

**Table 2:** Descriptive statistics and correlation analysis of variables.

Variable	M ± SD	1	2	3	4	5	6	7	8	9
1. Residence	1.41 ± 0.49	1								
2. Grade	1.95 ± 0.84	0.031	1							
3. Major	2.27 ± 1.36	−0.151**	−0.291**	1						
4. PS	2.76 ± 0.44	0.051	−0.007	0.053	1					
5. PE	13.55 ± 10.02	0.032	−0.156**	0.065	−0.120**	1				
6. NE	2.25 ± 0.55	0.006	−0.021	0.001	0.550**	−0.096*	1			
7. EE	2.70 ± 0.73	−0.013	−0.030	0.085*	0.259**	−0.095*	0.295**	1		
8. RE	2.51 ± 0.66	−0.067	0.035	0.050	0.227**	0.081*	0.396**	0.258**	1	
9. XE	3.27 ± 0.51	−0.022	0.044	0.036	0.105**	0.091*	0.109**	0.124**	0.087*	1
Kurtosis	—	—	—	—	0.463	1.274	−0.348	0.561	0.254	0.764
Skewness	—	—	—	—	−0.334	1.043	0.015	−0.159	−0.184	−0.146

*p < 0.05, **p < 0.01, ***p < 0.001, PS, perceived stress; PE, physical exercise; NE, negative emotions; EE, emotional eating; RE, restrained eating; XE, external eating.

### 3.2 Testing the mediating role of negative emotions

Following the recommendations of Wen et al. (32), in the first step, Model 4 of the PROCESS macro (Version 3.5) in SPSS 26.0 was employed to test the mediating effect of negative emotions between perceived stress and disordered eating after controlling for covariates including residence, grade and major. The results (see Table 3 and Figures 1–3) indicated that perceived stress showed a significant positive association with emotional eating, restrained eating, and external eating (b = 0.424, *p* < 0.001; b = 0.346, *p* < 0.001; b = 0.122, *p* < 0.05, respectively). Accordingly, research hypothesis 1 proposed in this study was supported. After incorporating negative emotions as a mediator, perceived stress still showed significant positive direct associations with emotional eating (b = 0.221, *p* < 0.01), whereas its direct association with restrained eating and external eating was not significant (b = 0.020, *p* > 0.05, b = 0.073, *p* > 0.05, respectively). Perceived stress showed a significant positive association with negative emotions (b = 0.696, *p* < 0.01). Negative emotions showed a significant positive association with emotional eating (b = 0.292, *p* < 0.001) and restrained eating (b = 0.469, *p* < 0.001), but its association with external eating was not significant (b = 0.070, *p* > 0.05).

**Table 3:** Regression coefficients from mediation models examining negative emotions as a mediator of perceived stress and three types of disordered eating.

Variable	NE		Model 1				Model 2				Model 3			
	b	t	b	t	b	t	b	t	b	t	b	t	b	t
Residence	−0.031	−0.790	−0.023	−0.397	−0.014	−0.250	−0.099	−1.837	−0.085	−1.666	−0.024	−0.554	−0.021	−0.504
Grade	−0.018	−0.767	−0.008	−0.215	−0.002	−0.069	0.040	1.219	0.048	1.565	0.035	1.368	0.036	1.418
Major	−0.016	−0.121	0.035	1.587	0.040	1.833	0.020	0.981	0.028	1.438	0.016	1.026	0.018	1.098
PS	0.696	16.190**	0.424	6.489***	0.221	2.873**	0.346	5.764***	0.020	0.296	0.122	2.576*	0.073	1.288
NE	—	—	—	—	0.292	4.779***	—	—	0.469	8.706***	—	—	0.070	1.565
R^2^	0.305		0.072		0.107		0.061		0.166		0.016		0.020	
F	65.633***		11.686***		14.258***		9.675***		23.865***		2.385*		2.402*	

The dependent variables for each model are Emotional Eating (Model 1), Restrained Eating (Model 2), and External Eating (Model 3), respectively. All regression coefficients presented are unstandardized coefficients (b). *p < 0.05, **p < 0.01, ***p < 0.001, PS, perceived stress; NE, negative emotions.

**Figure 1:**
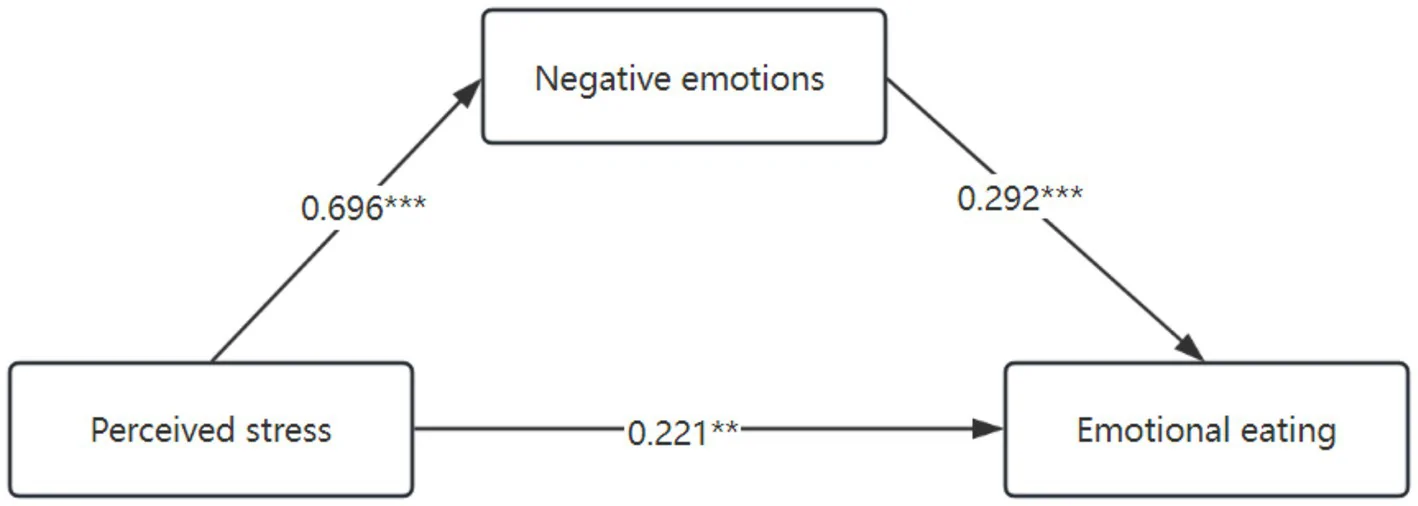
Mediation model 1 with emotional eating as the dependent variable.

**Figure 2:**
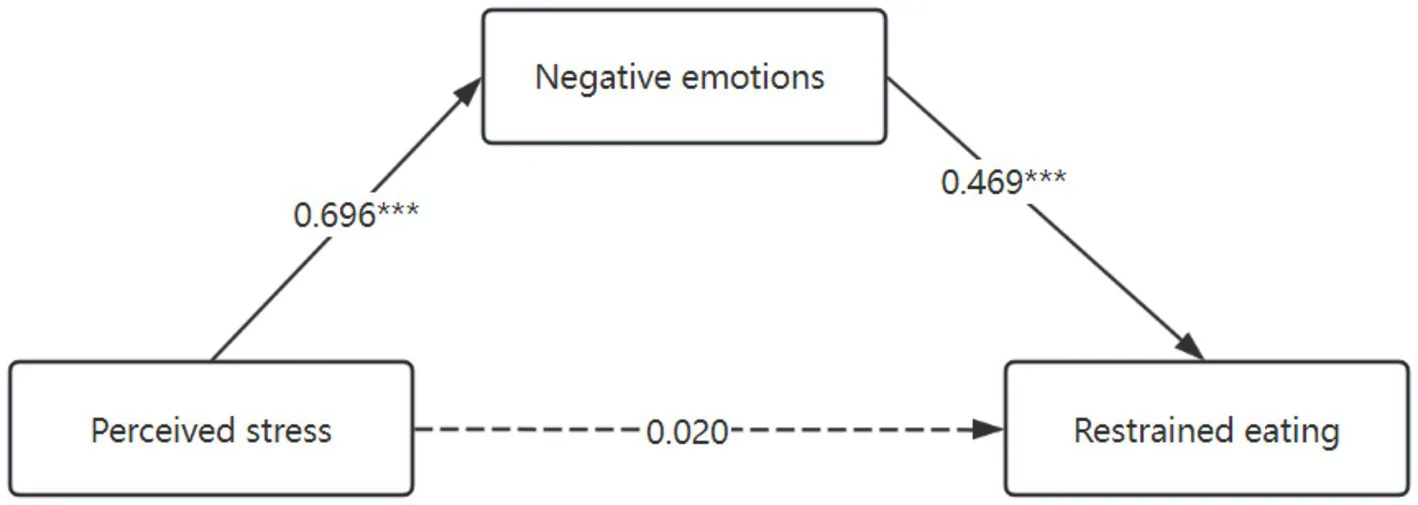
Mediation model 2 with restrained eating as the dependent variable.

**Figure 3:**
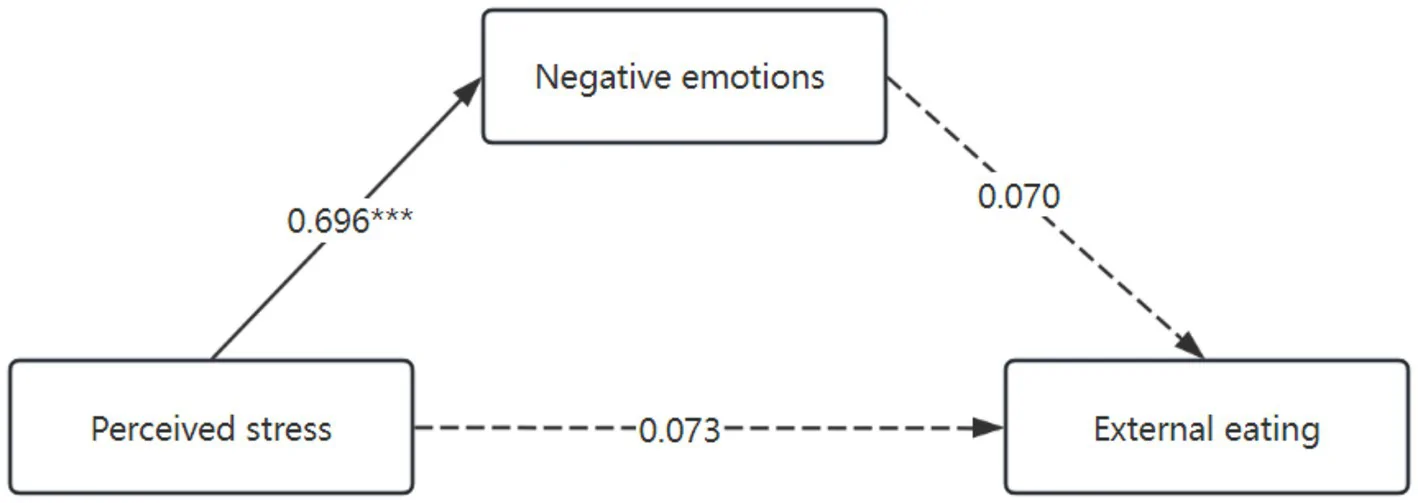
Mediation model 3 with external eating as the dependent variable.

As shown in Table 4, the indirect effect value of Mediation Path 1 was 0.203 (Boot SE = 0.047), with a 95% bootstrap confidence interval of [0.122, 0.294], which did not include zero. The indirect effect value of Mediation Path 2 was 0.326 (Boot SE = 0.045), with a 95% bootstrap confidence interval of [0.243, 0.418], which did not include zero. The indirect effect value of Mediation Path 3 was 0.049 (Boot SE = 0.035), with a 95% bootstrap confidence interval of [−0.017, 0.117], which included zero. The results of these path effect tests indicated that perceived stress was associated with emotional eating and restrained eating through the mediating role of negative emotions, whereas its association with external eating was not significant. Accordingly, research hypothesis 2 proposed in this study was partially supported.

**Table 4:** The mediating effects of negative emotions on the relationships between perceived stress and three types of disordered eating.

Dependent variable	Effect type	Path	Effect value	BootSE	Boot95%CI	
					Lower	Upper
EE	Total effect	—	0.424	0.070	0.283	0.565
	Direct effect	PS → EE	0.221	0.080	0.067	0.379
	Indirect effect	PS → NE → EE (Path 1)	0.203	0.047	0.122	0.294
RE	Total effect	—	0.346	0.062	0.222	0.465
	Direct effect	PS → RE	0.020	0.072	−0.124	0.162
	Indirect effect	PS → NE → RE (Path 2)	0.326	0.045	0.243	0.418
XE	Total effect	—	0.122	0.040	0.042	0.202
	Direct effect	PS → XE	0.073	0.054	−0.037	0.180
	Indirect effect	PS → NE → XE (Path 3)	0.049	0.035	−0.017	0.117

PS, perceived stress; NE, negative emotions; EE, emotional eating; RE, restrained eating; XE, external eating.

### 3.3 Testing the moderating role of physical exercise

In the second step, Model 59 of the PROCESS macro (Version 3.5) in SPSS 26.0 was employed to examine the moderating role of physical exercise in the relationships among perceived stress, negative emotions, and the three types of disordered eating, after controlling for covariates including residence, grade and major. The results (see Table 5) indicated that the interaction term of perceived stress and physical exercise showed a significant positive association with negative emotions (b = 0.010, *p* < 0.05) and emotional eating (b = 0.019, *p* < 0.05). However, the interaction term of perceived stress and physical exercise did not show a significant association with restrained eating (b = 0.011, *p* > 0.05) and external eating (b = 0.005, *p* > 0.05). Additionally, the interaction term of negative emotions and physical exercise showed a significant negative association with emotional eating (b = −0.016, *p* < 0.01) and restrained eating (b = −0.011, *p* < 0.05). However, the interaction term of negative emotions and physical exercise did not show a significant association with external eating (b = −0.004, *p* > 0.05). These findings suggested that physical exercise played a positive moderating role in the relationship between perceived stress and negative emotions and emotional eating, while it did not play a moderating role in the relationships between perceived stress and restrained eating and external eating. Physical exercise played a negative moderating role in the relationship between negative emotions and emotional eating and restrained eating, while it did not play a moderating role in the relationships between negative emotions and external eating. Thus, hypothesis 2 was partially supported.

**Table 5:** Moderating effects of physical exercise on the relationships between perceived stress, negative emotions, and three types of disordered eating.

Variable	Dependent variable							
	NE		EE		RE		XE	
	b	t	b	t	b	t	b	t
Residence	−0.029	−0.762	−0.010	−0.169	−0.093	−1.846	−0.027	−0.641
Grade	−0.019	−0.825	−0.010	−0.289	0.064	2.078*	0.047	1.828
Major	−0.014	−0.960	0.040	1.825	0.024	1.252	0.016	0.985
PS	0.680	15.613***	0.204	2.655**	0.038	0.566	0.085	1.504
PE	−0.001	−0.610	−0.004	−1.525	0.009	3.542***	0.006	2.874**
NE	—	—	0.304	4.952***	0.489	9.073***	0.079	1.744
PS × PE	0.010	2.100*	0.019	2.363*	0.011	1.476	0.005	0.813
NE × PE	—	—	−0.016	−2.667**	−0.011	−2.110*	−0.004	−0.895
R^2^	0.311		0.123		0.190		0.034	
F	44.865***		10.400***		17.410***		2.654**	

All regression coefficients presented are unstandardized coefficients (b). *p < 0.05, **p < 0.01, ***p < 0.001, PS, perceived stress; PE, physical exercise; NE, negative emotions; EE, emotional eating; RE, restrained eating; XE, external eating.

To further elucidate the moderating effects of physical exercise, this study divided participants into high and low physical exercise groups based on one standard deviation above and below the mean, and conducted simple slope analyses. The results (see Figures 4–7) revealed the following patterns. In the relationship between perceived stress and negative emotions and emotional eating: For college students with higher levels of physical exercise (M + 1SD), perceived stress showed a significant positive association with negative emotions (b = 0.775, *p* < 0.001) and emotional eating (b = 0.399, *p* < 0.001); for those with lower levels of physical exercise (M–1SD), perceived stress also showed a significant positive association with negative emotions (b = 0.584, *p* < 0.01) and emotional eating (b = 0.009, *p* > 0.05), but with a smaller slope value. This indicated that as physical exercise levels increased, the positive associations of perceived stress with negative emotions and emotional eating gradually strengthened.

**Figure 4:**
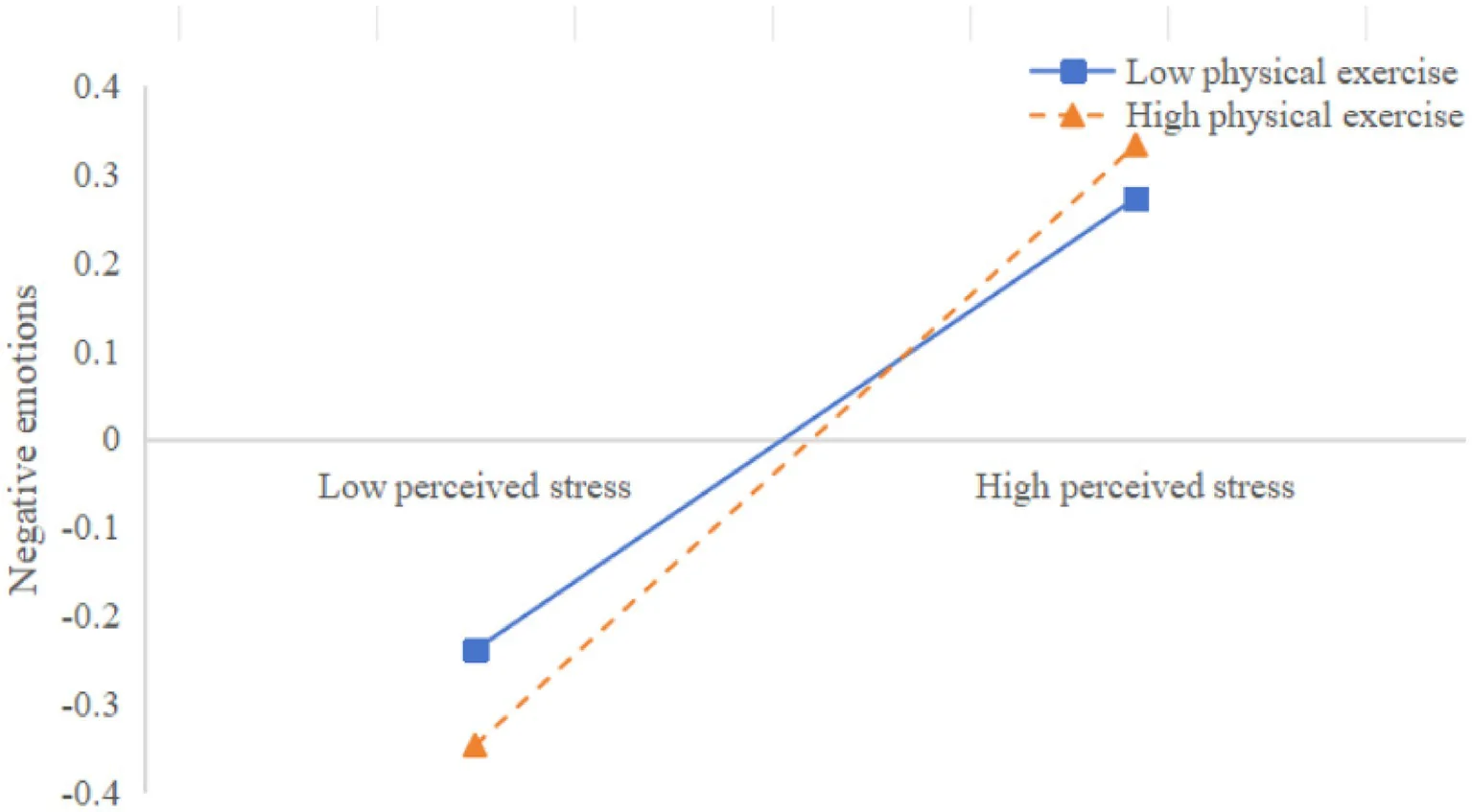
Simple slope analysis: the moderating effect of physical exercise on the relationship between perceived stress and negative emotions.

**Figure 5:**
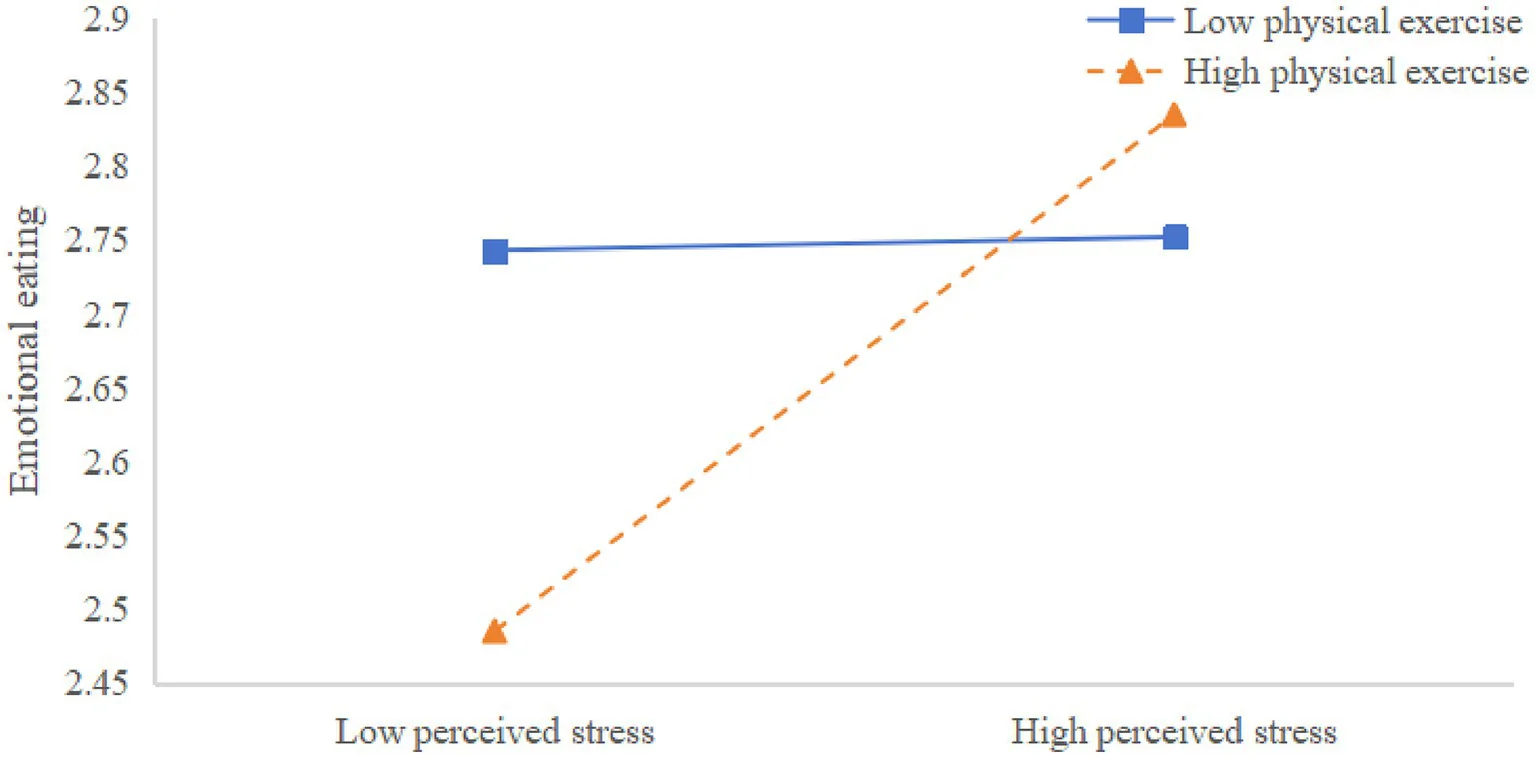
Simple slope analysis: the moderating effect of physical exercise on the relationship between perceived stress and emotional eating.

**Figure 6:**
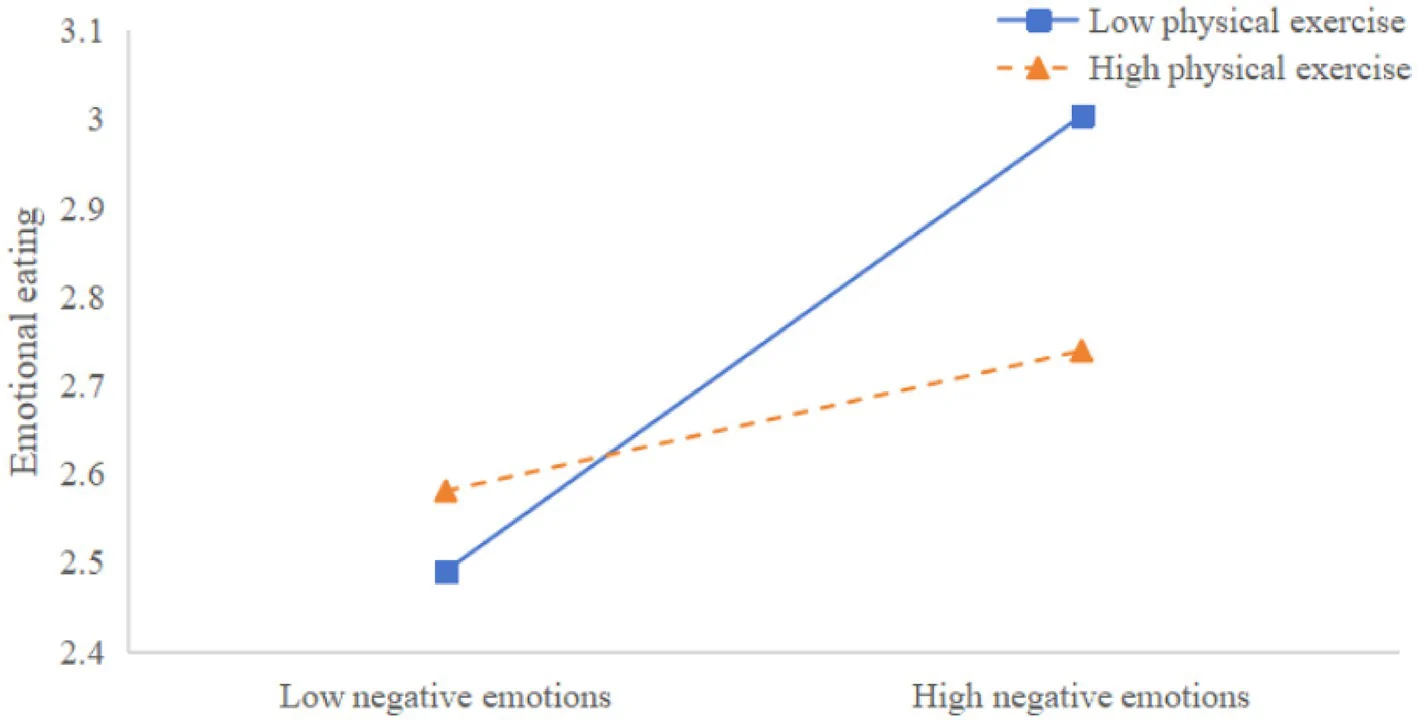
Simple slope analysis: the moderating effect of physical exercise on the relationship between negative emotions and emotional eating.

**Figure 7:**
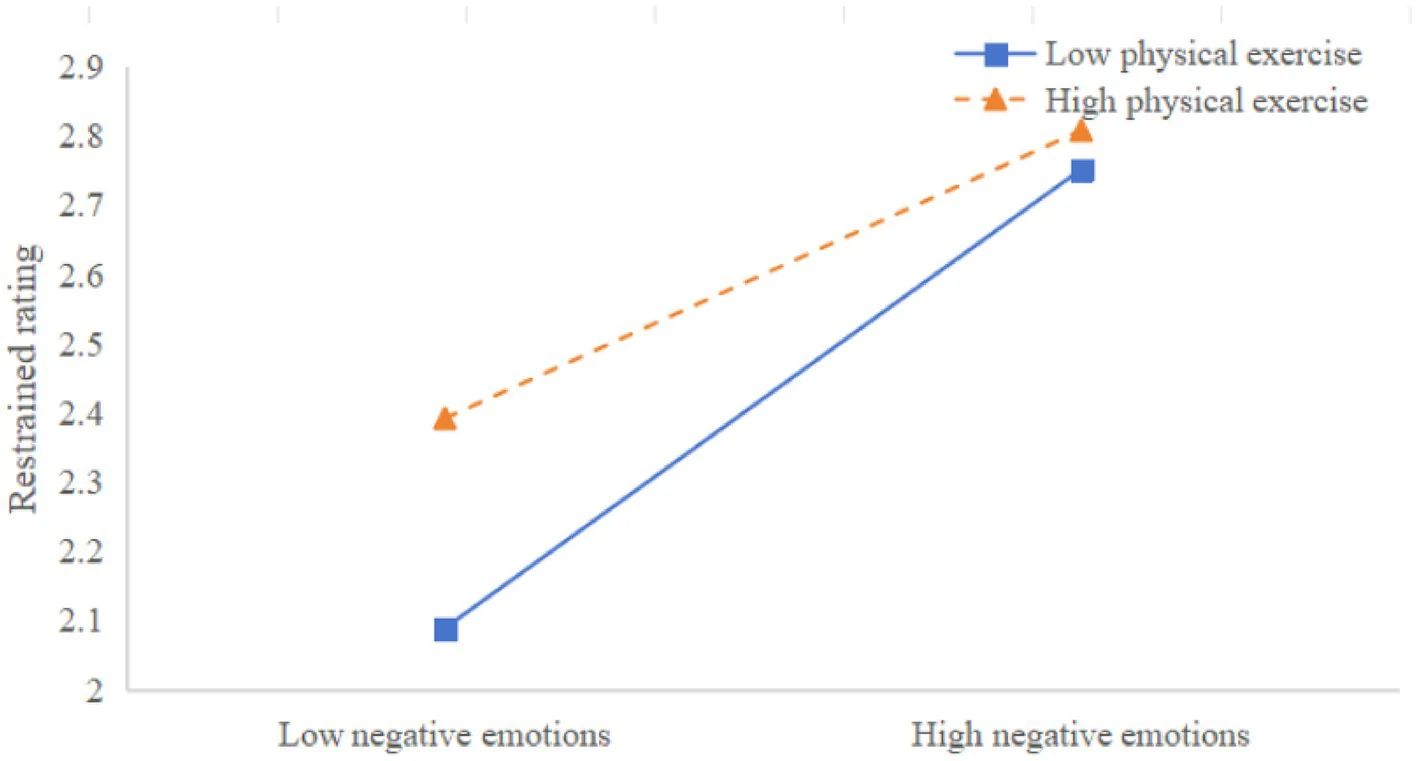
Simple slope analysis: the moderating effect of physical exercise on the relationship between negative emotions and restrained eating.

In the relationships between negative emotions and emotional eating and restrained eating: For college students with higher levels of physical exercise (M + 1SD), negative emotions showed a significant positive association with negative emotional eating (b = 0.143, *p* > 0.05) and restrained eating (b = 0.378, *p* < 0.001); for those with lower levels of physical exercise (M–1SD), negative emotions also showed a significant positive association with emotional eating (b = 0.465, *p* < 0.001) and restrained eating (b = 0.601, *p* < 0.001), but with a larger slope value. This indicated that as physical exercise levels increased, the positive association of negative emotions with emotional eating and restrained eating gradually weakened.

## 4 Discussion

This study focused on female college students as the primary research population to examine the associations among perceived stress, negative emotions, and disordered eating (i.e., emotional eating, restrained eating, and external eating), with particular attention to the moderating role of physical exercise in these associations. The following sections provide a detailed analysis of the associations among the aforementioned variables.

### 4.1 Analysis of the association between perceived stress and disordered eating

This study found that, without considering other variables, perceived stress showed a significant positive association with all three dimensions of disordered eating (i.e., emotional eating, restrained eating, and external eating). This finding is not only consistent with previous research (33) but also in line with the fundamental premise of stress-coping theory, which posits that individuals often resort to disordered eating as a means of alleviating maladaptive psychological responses when confronted with stressful situations (34, 35). However, it is noteworthy that after incorporating negative emotions as a mediator, the patterns of association between perceived stress and the three dimensions of disordered eating diverged. Specifically, perceived stress still showed a significant positive direct association with emotional eating, whereas its direct associations with restrained eating and external eating were not significant. This pattern suggests that negative emotions play distinct mediating roles across the three types of disordered eating. The detailed mediating effects are further examined in the following section (4.2).

### 4.2 Analysis of the mediating role of negative emotions

This study found significant differences in the mediating roles of negative emotions between perceived stress and the three dimensions of disordered eating among female college students. Specifically, the indirect effect was strongest for restrained eating, followed by emotional eating, whereas the indirect effect for external eating was not significant. This variability indicates that the mediating role of negative emotions in the relationship between perceived stress and disordered eating depends on the type of eating behavior. For emotional eating, the indirect effect via negative emotions was significant, while the direct effect of perceived stress remained significant after including the mediator. This partial mediation pattern suggests that perceived stress influences emotional eating both directly and indirectly through the elicitation of negative emotions (36, 37). This finding implies that stress may be related to emotional eating not only by triggering negative affect but also through other direct pathways (35). From an intervention perspective, this suggests that efforts to address emotional eating should focus not merely on reducing stressors but also on enhancing individuals’ capacity to tolerate and regulate negative emotions (38).

In contrast, for restrained eating, the indirect effect via negative emotions was significant, whereas the direct effect of perceived stress became non-significant after including the mediator. This full mediation pattern indicates that the association between perceived stress and restrained eating operates entirely through negative emotional experiences. Specifically, stress-related negative emotions may intensify concerns about weight and body shape among female college students during low-mood states (39), thereby promoting restrained eating tendencies. Unlike emotional eating, which retains a direct pathway from stress, restrained eating appears to be more purely emotion-driven in its linkage to stress, with no significant stress-to-behavior pathway independent of negative emotions.

For external eating, the indirect effect via negative emotions was not significant, nor was the direct effect of perceived stress. This indicates that negative emotions do not mediate the relationship between perceived stress and external eating, suggesting that the mechanisms underlying external eating may be fundamentally distinct from emotion-driven pathways. Rather than reflecting internal emotional states, external eating is more likely to involve heightened sensitivity to external food cues (e.g., sight, smell, or palatability of food) and diminished inhibitory control over such cues (40, 41). Future research could link external eating to phenomena such as binge eating and food addiction to explore its automatic activation mechanisms under external environmental stimuli.

### 4.3 Analysis of the moderating role of physical exercise

This study found that physical exercise played a positive moderating role in the relationship between perceived stress and negative emotions; that is, higher levels of physical exercise were associated with a stronger association between stress and negative emotions. This finding appears to contradict conventional wisdom, which generally holds that physical exercise buffers the negative effects of stress (42, 43). However, it should be noted that although this moderating effect reached statistical significance (b = 0.010, *p* < 0.05), the regression coefficient was very small, suggesting that the practical real-world significance of this weak effect size is limited. With this caveat in mind, several possible explanations may still be considered. From the perspective of interoceptive awareness theory, individuals who regularly engage in physical exercise may develop heightened sensitivity to their own psychophysiological states (44). This enhanced interoceptive awareness may lead them to more accurately perceive or even overestimate stress-related bodily signals, thereby reporting higher levels of perceived stress and negative emotions on subjective measures (45, 46). This does not necessarily indicate that these individuals are experiencing more severe actual stress; rather, it may reflect differential sensitivity to internal cues among individuals with higher levels of body–mind awareness. Furthermore, from the perspective of ego-depletion theory, individuals who regularly participate in physical exercise may deplete substantial psychological resources (e.g., self-discipline, persistence, time management) (47), making them more susceptible to resource exhaustion when facing additional stressors and, consequently, more prone to emotional distress than individuals with lower resource investment (48). Nevertheless, given the minimal magnitude of this moderating effect, these theoretical interpretations should be treated with caution, and the finding warrants further replication in future studies.

In addition, physical exercise also exerted a positive moderating effect on the direct relationship between perceived stress and emotional eating. That is, higher levels of physical exercise were associated with a stronger direct association between perceived stress and emotional eating. This finding appears counterintuitive, as physical exercise might be expected to weaken, rather than strengthen, the stress-eating link (49, 50). One possible explanation is that individuals who exercise regularly may possess heightened body awareness and are therefore more attuned to stress-induced physiological changes, which could amplify their perceived need to eat in response to stress (44, 46). Alternatively, from a behavioral consistency perspective, individuals with high physical exercise engagement may hold strong beliefs about health and body management (51). When stress disrupts their regular exercise routines or challenges their health-related self-concept, this dissonance may paradoxically increase their inclination toward emotional eating as a compensatory coping response (52). Nevertheless, given the small magnitude of this moderating effect, these interpretations remain speculative and require further empirical validation.

Notably, physical exercise played a negative moderating role in the relationships between negative emotions and both emotional eating and restrained eating. Specifically, as levels of physical exercise increased, the positive associations of negative emotions with emotional eating and restrained eating gradually weakened. This indicates that physical exercise can indeed serve as a “buffering barrier” between negative emotions and disordered eating (53), interrupting the chain from “negative mood → disordered eating.” The mechanism underlying this moderating effect may be related to improvements in prefrontal executive functions resulting from physical exercise (54). Relevant studies have consistently demonstrated that regular aerobic exercise is associated with enhanced cognitive control functions of the prefrontal cortex, including inhibitory control, emotion regulation, and working memory updating (54, 55). When negative emotions arise, individuals with well-developed executive functions are better able to inhibit impulsive eating tendencies and adopt more adaptive emotion regulation strategies as alternatives to disordered eating (52). Thus, the protective role of physical exercise may not act directly on emotions themselves but rather may operate by strengthening cognitive control resources, thereby blocking the pathway from emotional experience to maladaptive eating behaviors.

However, the moderating effect of physical exercise on the relationship between negative emotions and external eating was not significant. This is consistent with the earlier finding that external eating is largely driven by external food cues rather than internal emotional states (56). As a behavior primarily triggered by environmental stimuli (e.g., the sight or smell of palatable food), external eating may be less susceptible to emotion-focused interventions such as physical exercise. This also aligns with the dual-system model of eating behavior, which posits that the endogenous emotional system and the exogenous environmental system influence eating behaviors through relatively independent pathways (57). For external eating, interventions targeting environmental cue control (e.g., stimulus exposure reduction, mindful eating training) may be more effective than those primarily aimed at emotional regulation.

Additionally, physical exercise did not exert a significant moderating effect on the relationships between perceived stress and restrained eating or perceived stress and external eating. This suggests that the buffering role of physical exercise may be more salient in the emotion-to-behavior stage (i.e., NE → disordered eating) than in the stress-to-emotion stage (i.e., PS → NE), and is primarily effective for emotionally driven eating patterns (i.e., emotional eating and restrained eating). In other words, physical exercise may be better understood as a “downstream” protective factor that disrupts the translation of negative affect into eating actions, rather than as an “upstream” factor that prevents the generation of negative emotions from stress. Accordingly, research hypothesis 2 was partially supported.

### 4.4 Limitations and implications

First, this study employed a cross-sectional design, which precludes making strong causal inferences about the associations among variables. Future research should adopt longitudinal tracking designs or experimental studies to verify causal directions. Second, all data were derived from self-report questionnaires, which may be subject to common method bias and social desirability bias. Although Harman’s single-factor test indicated that common method bias was not severe, self-report data may still be influenced by recall bias and subjective judgment bias. Future studies could consider incorporating objective indicators (e.g., cortisol levels, body composition measurements) or other-report data to enhance the reliability of findings. Third, the sample of this study was limited to female college students; whether the findings can be generalized to male college students or other age groups requires further verification. Gender differences play significant roles in stress coping, emotion regulation, and eating behaviors (52), and future research should include male samples for comparative analysis. Finally, this study did not conduct subgroup analyses (e.g., by university, grade, or major) due to the primary focus on examining the overall mediating and moderating effects among the core psychological variables, and the sample size did not provide sufficient statistical power for reliable subgroup comparisons. Future research with larger and more diverse samples could explore whether the observed relationships vary across different institutional contexts, academic years, or majors.

The above findings offer several implications for mental health and disordered eating interventions among female college students. First, given that negative emotions partially mediate the relationship between perceived stress and emotional eating, interventions for emotional eating should prioritize emotion regulation training while also addressing stress management directly, as the direct effect of stress remains significant. For restrained eating, which is fully mediated by negative emotions, interventions should primarily focus on emotional awareness and tolerance, with cognitive restructuring around body image as a secondary component given the heightened weight and shape concerns prevalent in this population. Second, physical exercise is recommended as an effective means of mitigating the transition from negative emotions to emotionally driven eating patterns (i.e., emotional eating and restrained eating), as it was found to weaken the positive associations between negative emotions and these two types of disordered eating. However, the practical utility of physical exercise in preventing stress-induced emotional distress should not be overstated among female college students, as its positive moderating effects on the stress-emotion pathways were statistically significant but very small in magnitude. Third, external eating requires distinct approaches focusing on environmental control strategies, such as exposure and response prevention to external food cues, rather than emotion-focused interventions, as it is neither directly nor indirectly associated with stress or negative emotions.

## 5 Conclusion

In conclusion, this study found that negative emotions served as a partial mediator between perceived stress and emotional eating, a full mediator between perceived stress and restrained eating, and did not mediate the relationship with external eating among female college students. Physical exercise negatively moderated the associations between negative emotions and both emotional eating and restrained eating, suggesting its protective role in interrupting the emotion-to-behavior pathway, though this moderating effect did not extend to external eating. Additionally, physical exercise positively moderated the stress–negative affect pathway, but the small effect sizes indicate that this finding should be interpreted with caution and its practical significance is limited.

## Data Availability

The original contributions presented in the study are included in the article/supplementary material, further inquiries can be directed to the corresponding author.
